# Updating the evidence base on the operational costs of supplementary immunization activities for current and future accelerated disease control, elimination and eradication efforts

**DOI:** 10.1186/1471-2458-14-67

**Published:** 2014-01-22

**Authors:** Gian Gandhi, Patrick Lydon

**Affiliations:** 1 United Nations Children’s Fund, 3 United Nations Plaza, New York, NY 10017, USA; 2 World Health Organization, 20 Avenue Appia, CH-1211, Geneva 27, Switzerland

## Abstract

**Background:**

To achieve globally or regionally defined accelerated disease control, elimination and eradication (ADC/E/E) goals against vaccine-preventable diseases requires complementing national routine immunization programs with intensive, time-limited, and targeted Supplementary Immunization Activities (SIAs). Many global and country-level SIA costing efforts have historically relied on what are now outdated benchmark figures. Mobilizing adequate resources for successful implementation of SIAs requires updated estimates of non-vaccine costs per target population.

**Methods:**

This assessment updates the evidence base on the SIA operational costs through a review of literature between 1992 and 2012, and an analysis of actual expenditures from 142 SIAs conducted between 2004 and 2011 and documented in country immunization plans. These are complemented with an analysis of budgets from 31 SIAs conducted between 2006 and 2011 in order to assess the proportion of total SIA costs per person associated with various cost components. All results are presented in 2010 US dollars.

**Results:**

Existing evidence indicate that average SIA operational costs were usually less than US$0.50 per person in 2010 dollars. However, the evidence is sparse, non-standardized, and largely out of date. Average operational costs per person generated from our analysis of country immunization plans are consistently higher than published estimates, approaching US$1.00 for injectable vaccines. The results illustrate that the benchmarks often used to project needs underestimate the true costs of SIAs and the analysis suggests that SIA operational costs have been increasing over time in real terms. Our assessment also illustrates that operational costs vary across several dimensions. Variations in the actual costs of SIAs likely to reflect the extents to which economies of scale associated with campaign-based delivery can be attained, the underlying strength of the immunization program, sensitivities to the relative ease of vaccine administration (i.e. orally, or by injection), and differences in disease-specific programmatic approaches. The assessment of SIA budgets by cost component illustrates that four cost drivers make up the largest proportion of costs across all vaccines: human resources, program management, social mobilization, and vehicles and transportation. These findings suggest that SIAs leverage existing health system infrastructure, reinforcing the fact that strong routine immunization programs are an important pre-requisite for achieving ADC/E/E goals.

**Conclusions:**

The results presented here will be useful for national and global-level actors involved in planning, budgeting, resource mobilization, and financing of SIAs in order to create more realistic assessments of resource requirements for both existing ADC/E/E efforts as well as for new vaccines that may deploy a catch-up campaign-based delivery component. However, limitations of our analysis suggest a need to conduct further research into operational costs of SIAs. Understanding the changing face of delivery costs and cost structures for SIAs will continue to be critical to avoid funding gaps and in order to improve vaccination coverage, reduce health inequities, and achieve the ADC/E/E goals many of which have been endorsed by the World Health Assembly and are included in the Decade of Vaccines Global Vaccine Action Plan.

## Background

Over the past three decades, several global health initiatives have brought together public and private stakeholders to focus efforts on achieving globally or regionally defined goals of accelerated disease control^a^, elimination and eradication (ADC/E/E) of several vaccine-preventable diseases—notably poliomyelitis (polio), measles/rubella, maternal and neonatal tetanus (MNT), yellow fever, and *Neisseria meningitis* serogroup A (menA) [1-8]. These initiatives have combined strategic focus, global coordination, expertise, and capacity to assist country governments mainly in low income countries (LICs) and middle income countries (MICs), complementing national routine immunization activities with intensive, time-limited, and targeted vaccination campaigns – Supplementary Immunization Activities (SIAs) [9-11]. At best, well-funded SIAs represent an important delivery strategy to improve population immunity, reduce inequities in access to vaccination, and help achieve ambitious public health goals.

Prices of the vaccines used in ADC/E/E initiatives have reached maturity in that they are relatively affordable (i.e. roughly $0.50 per dose or less) and in most cases supply and prices are quite stable [12]. From a funding standpoint, the cost of these vaccines have historically been covered by global financers of immunization such as the GAVI Alliance (formerly the ‘Global Alliance for Vaccines and Immunizations’); or vaccine costs have been covered by the resources mobilized by the partnerships that have coalesced around ADC/E/E efforts like the Global Polio Eradication Initiative (GPEI) and the Measles-Rubella Initiative (MRI). However, since the late 1990s, the increasing number of ADC/E/E as well as other health priorities has increased competition for resources. Funding from both national and global sources has often been insufficient to cover vaccine and non-vaccine SIA costs, but particularly the operational costs of SIAs. Along with a variety of other factors, funding shortfalls have led to delays in implementation or forced SIA efforts to be scaled back (i.e. to target narrower age groups) resulting in campaigns not reaching the necessary coverage rates nor the levels of population immunity necessary for disease control, in some cases resulting in large disease outbreaks after re-introduction of virus [13-16].

SIA funding shortfalls are likely to have been precipitated by a number of factors: ADC/E/E programs essentially deliver a ‘global good’ whose benefit may be underestimated on a national basis leading to underinvestment by national payers. The somewhat unpredictable nature of disease dynamics associated with many of the viruses targeted by ADC/E/E efforts may make longer term planning and budgeting for SIAs difficult at the national level. The broad age ranges targeted particularly with catch-up SIAs, and to a lesser extent follow-up SIAs, is likely to stretch national health budgets particularly where external partner funding (e.g. from GAVI Alliance or MRI) is not available. While weak governance within many of the countries that are worst affected by these diseases almost certainly limits the effectiveness of monies earmarked for SIAs.

Combining the above factors with the lack of information about the real operational costs of SIAs, how these costs differ by disease, delivery strategy, and setting, and how they may be changing over time, may have hampered the ability of payers at both country- and global levels to allocate adequate funds to cover SIA costs.

Looking ahead and beyond the existing ADC/E/E initiatives, it is clear that over the coming decade SIAs will continue to be an integral part of vaccination delivery strategies in combination with routine vaccine delivery (through outreach and fixed site –based approaches). Several underutilized and new vaccines that seem likely to be adopted across LICs and MICs in the coming years will include an SIA delivery component: either a one-time catch up campaign at the time of introduction to accelerate disease control (i.e. Japanese Encephalitis (JE) vaccines, typhoid conjugate vaccines), or periodic campaigns to maintain herd immunity (i.e. cholera vaccines) [17]. For these newer or still underutilized vaccines, all of which are components of the global immunization agenda set out in the Decade of Vaccines (DoV) Global Vaccine Action Plan (GVAP), there is limited evidence on the costs of campaign-based delivery making it more difficult to predict the necessary resource requirements to achieve the DoV goals [18-22]. Until such tailored evidence can be generated, the most up-to-date analogous evidence from existing ADC/E/E initiatives will be critical for long range financial planning.

### Objectives

The objectives of the work described in this paper are to:

● review published and grey literature on the operational costs associated with implementation of SIAs and summarize the results;

● estimate the average operational costs associated with implementation of SIAs based on actual costs reported by countries;

● compare older estimates of operational costs (from 2004–2008) from literature to more recent estimates (from 2009–2011);

● explore how average operational costs vary by vaccine, geography, and program;

● assess the main cost components of operational costs from available budget data; and to

● discuss the policy and practice implications of these findings for future SIA efforts.

## Methods

The research was divided into two parts – (I) A literature review; and (II) An analysis of data from country-specific and global immunization plans and budgets.

### (I)→Literature review

A review of the relevant literature, both the peer-reviewed and non-peer reviewed grey literature, between 1992 and 2012 was conducted using the PubMed database with the aim of identifying SIA operational costs estimates and expenditures. The review used the following inclusion criteria:

● Paper must deal with an SIA to deliver human vaccines rather than veterinary vaccines;

● Paper must deal with an SIA conducted in an LIC or MIC;

● Paper’s emphasis must be on *SIAs/mass vaccination campaigns* rather than c*ommunications/media campaigns* that might be related to vaccination efforts;

● Paper must include primary source data on the operational costs of SIAs;

● Paper must include operational cost data that can be separated from the costs of vaccines, and expressed on a per person targeted/vaccinated basis^b^;

● Paper must have an available English language abstract.

Search terms included: “supplement* immunization activity cost”, “SIA cost”, “vaccin* campaign cost”, “mass vaccin* cost”, “vaccin* operational cost”, “polio eradication cost”, “measles elimination cost”, “vaccin* disease control cost”. Spelling variations and permutations of these search terms were also included.

To ensure comparability, we specified all operational costs on a per targeted person per dose basis to control for differences in vaccination course. Where possible we attempted to control for differences in costing definitions, in particular excluded opportunity costs, indirect costs, the costs of vaccines, injection supplies, freight and customs charges (for international shipment of vaccines), and international technical assistance to support the SIAs including agency overhead costs. We also controlled for temporal distortions by inflation adjusting cost estimates into 2010 dollars using published deflators and converting costs from local currencies into United States dollars (US$) using published historic exchange rates [23,24]. Finally, we drew upon and extended a previously published framework as a template for summarizing papers containing SIA operational costs [25].

### (I)→Analysis of data from country-specific plans – See Additional file 1

#### Analysis description

A previous unpublished analysis of costed country immunization plans assessed the operational costs per person for many of the vaccines noted above (P. Lydon and G. Gandhi: *Introduction of New Vaccines: Analysis of non-vaccine routine and campaign costs for the GAVI Alliance.* Unpublished). The previous analysis was based on a heterogeneous dataset that included actual costs and expenditures as well as projected costs. Since projections often rely on out of date benchmarks or historic expenditures, the unpublished study may have underestimated the true operational costs of SIAs. In this paper, we update the analysis restricting to a more homogenous and valid dataset (discussed in more detail below in the *Analytic approach*), and extend the analysis to illustrate how per person operational costs varied in aggregate by:

(i) Temporal characteristics – to assess how costs have changed over time.

● Comparing estimates identified in the literature with those generated from comprehensive multi-year plans (cMYPs) data.

● Comparing older estimates (from 2004–2008) from cMYPs versus more recent estimates (from 2009–2011) in aggregate across the dataset, as well as on a country-by-country basis where a country has conducted two SIAs against the same vaccine-preventable in the two time periods.

(ii) Vaccine delivery characteristics – to assess the effects on costs of different target populations and methods of administration.

● Comparing Oral Polio vaccines (OPV) and measles vaccines both targeting mainly under-five populations or slightly older children (in the case of measles vaccines), and tetanus-containing vaccines (e.g. tetanus toxoid—TT, tetanus-diphtheria—Td vaccines) targeting at-risk women of child bearing age (WCBA) populations^c^.

● Comparing interventions administered orally^d^ versus injectable vaccination.

(iii) Country characteristics – to assess the effects of population size, region, and program strength.

● Comparing two country groupings by population size: Midsize/Large Countries with total population ≥ 10 million, and Small Countries with total population <10 million)^e^.

● Comparing geographic regions as defined by the World Health Organization (WHO) regional groupings [26].

● Comparing the underlying strength of the routine immunization program, using estimated levels of vaccination coverage at the time the SIA was conducted, as a proxy (Grouping countries with coverage ≤ 70%, countries with coverage between 71% and 89%, and countries with coverage≥ 90%)^f^.

We also analyzed the operational costs across the portfolio of vaccines that have a campaign-based delivery component (i.e. JE, measles, measles-rubella (MR), menA, typhoid, and yellow fever vaccines) and that the GAVI Alliance supports or has committed to support [27]. In particular, we sought to assess how the actual operational cost expenditures compare to the levels of support that GAVI offers to countries conducting SIAs; namely US$0.30 per targeted person for SIAs conducted between 2004 and 2012, and US$0.65 per targeted person thereafter [28].

#### Data sources

The primary source for this analysis was country-specific data from cMYPs submitted to the WHO and the United Nations Children’s Fund (UNICEF) in 2009 and 2011 [29]. cMYPs are used mainly by immunization program managers in LICs and MICs to articulate a long-term costed plan for a national immunization program. For the reference-style data underlying the analysis (e.g., country population size data, GDP deflators), we employed datasets validated by third-party multilateral agencies [30,31].

#### Analytic approach

Each cMYP records actual cost data for a ‘baseline year’, followed by a five-year projection period in which future costs of the program are estimated. We extracted and aggregated operational cost data and total target population data per SIA conducted from the cMYP baseline years alone in order to focus on the best quality and most homogeneous actual information^g^. The cost data were consolidated and inflation-adjusted into 2010 dollars [23]. The inflation-adjusted operational costs per SIA were then combined with data on relevant target populations specified in the cMYP in order to control for the different target populations of campaigns and to generate standardized operational costs metrics on a population-weighted per person basis.

#### Sample descriptive statistics

**Figure 1:**
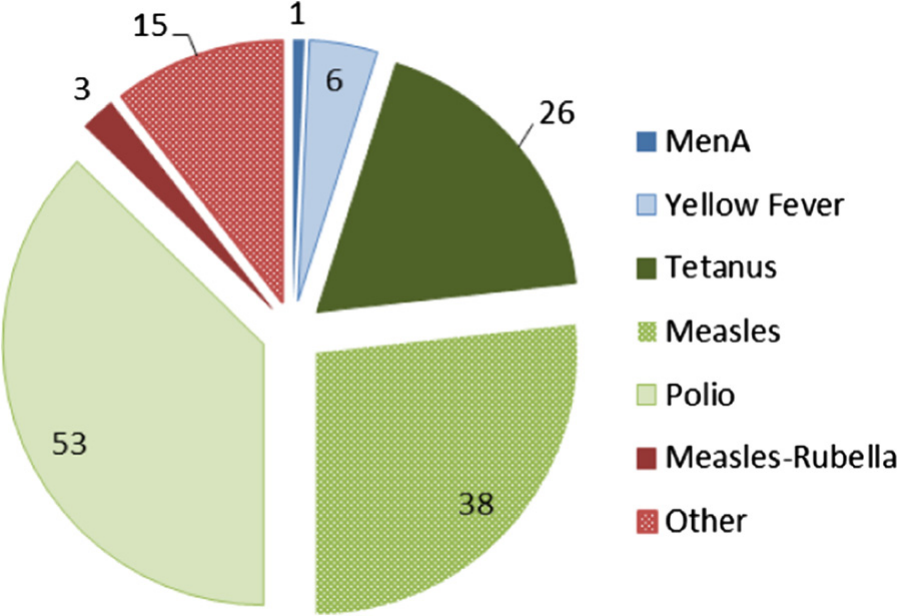
Distribution of SIA expenditure estimates by vaccine-preventable disease, 2004–2011 – cMYP data (N=142).

The extracted baseline cMYP data constitutes 142 SIA country observations of SIA operational costs from 70 cMYPs produced by 40 countries; i.e. several countries developed more than one cMYP, and several countries conducted more than one SIA over the analytical period. Of the 142 observations, 75 are from the period 2004–2007 while 67 are from the period 2008–2011. The distribution of the data by vaccine is illustrated in Figure 1.

In terms of country size and geographic location, 64% of the SIA observations were for Midsize/Large countries, versus 36% for Small countries. Of the observations, 66% were for SIAs conducted in the African region (AFR), 21% in the Eastern Mediterranean (EMR), 11% in South East Asia (SEAR), and the remaining 2% in Western Pacific (WPR) and European (EUR) regions [26]. There were no SIA observations for the Americas region (AMR) because cMYPs are not produced in the same format for countries in this region. Given this regional distribution, only the average operational costs for AFR, EMR and SEAR are presented here since WPR/EUR observations were insufficient to draw out any meaningful results. In terms of immunization program capacity/performance as measured by vaccination coverage, 23% of all the country observations were from countries with DTP3 ≤ 70% at the time the SIA was conducted, 45% from countries with DTP3 between 71% and 89%, and 32% from countries with DTP3 ≥ 90%. By contrast, using MCV1 coverage, the distribution of countries is markedly different; 49% of countries had MCV1 ≤ 70% at the time the SIA was conducted, 32% from countries with MCV1 between 71% and 89%, and 19% from countries with MCV1 ≥ 90%. Finally, in terms of country wealth as measured by per capita income, the vast majority of countries (71%) were classified as Low Income Countries (LICs) by the World Bank at the time of conducting the SIAs, while the remainder of the sample was classified as Lower Middle Income Countries (LMICs).

### (I)→Analysis of data from country-specific budgets – See Additional file 1

#### Analysis description

Separate from the interrogation of *expenditures* described above, an analysis of SIA *budgets* was undertaken to understand the main cost components of campaign operational costs. Budget data on operational costs was assessed in aggregate by Vaccine delivery characteristics. The sample size was too small to enable meaningful assessment of the data by temporal or country characteristics (although the planned timing of the campaigns and country characteristics are noted in Additional file 1).

#### Data sources

Budget data were used for the analysis of operational cost components because cMYP data do not disaggregate operational cost and expenditure information by cost component. SIA budgets came from a variety of sources: Budget breakdowns are requested as part of previous GAVI New Vaccine Support (NVS) application forms so these were used largely to provide a picture of menA and yellow Fever SIA operational cost components [32]. In addition, SIA budget information is often shared with ADC/E/E program coordinators at WHO, UNICEF and other technical agencies involved in these initiatives and particularly for measles and tetanus SIAs. For the reference-style data, we again employed datasets validated by third-party multilateral agencies [30,31].

#### Analytic approach

For the analysis of operational cost components, the various campaign budgets from 31 SIAs conducted between 2006 and 2011 were coded into a standardized format, and inflation-adjusted into 2010 dollars. These data were then analyzed to assess the proportion of total SIA costs per person for each cost component.

#### Sample descriptive statistics

The campaign budget analysis relied on a much smaller sample – 31 SIAs, of which 32% were for measles vaccines, 29% Tetanus, 26% menA, and 13% yellow fever. The majority (68%) of these SIAs were planned to take place in countries in the AFR region, most (77%) in mid-sized or large countries (i.e. countries with total populations >10 million), and most (58%) in countries with MCV1 between 71% and 89%. Equally, most (58%) of the budgets were for LICs.

## Results and discussion

### (I)→Literature review

Searches yielded 394 unique results. Of these, 381 were excluded following review of their abstracts because they were found not to meet the inclusion criteria – The remaining 13 manuscripts were then reviewed and pertinent information was extracted. Four further citations of papers/unpublished reports not identified in the initial searches were retrieved and assessed against the inclusion criteria. Overall, 17 papers and reports met the inclusion criteria.

Table 1 illustrates the summary findings from the remaining papers. Outside of measles vaccine SIAs, data is sparse, and surprisingly so for OPV and TT SIAs given their frequency. The vast majority of estimates come from studies conducted 10+ years ago, and mainly in the African region. All operational costs per person per dose were in the region of US$0.50 (median: $0.33; range: $0.03-$0.79) and all under US$1.00.

**Table 1:** High-level summary of literature on the operational costs of SIAs

**Reference**	**Vaccine/intervention details**	**Country/setting**	**Target population**	**Perspective**	**Cost data collection**	**Average versus Incremental costs**	**Year of cost data**	**Inflation-adjusted ops cost per person per dose, US$**	**Comparable inflation-adjusted cMYP estimate, US$ (Year of cMYP data)**	**Cost components included**
[33]	Measles vaccine	Catch up SIA in Afghanistan	Children, 6 mnths - 12 yrs.	External donor	Retrospective	Incremental	2002	$0.194	N/A	Training, incentives (per diems), transport, monitoring, social mobilization, and logistics
		Follow up SIA in Afghanistan	Children aged 6–59 mnths				2003	$0.244	$0.33 (2009)	
[34]	Measles vaccine	Nationwide SIA in Zambia	Children, 9 mnths −4 yrs.	National payer	Retrospective	Incremental	2001	$0.028	$0.73 (2010)	Social mobilization, supervision, planning/training, administrative costs, additional transport, additional personnel
Tsogbe K: Measles Campaign CEA, Rwanda. Unpublished.	Measles vaccine	Nationwide SIA in Rwanda	Children, 6–59 mnths	National payer and External donor	Retrospective	Average	2001	$0.284	$0.41 (2005)	Micro- planning, social mobilization, training, transport, fuel, and per diems (defined as "operational costs")
[35]	Measles vaccine	Pilot SIA in 2 provinces in Lao PDR	Children, 6–59 mnths	National payer and External donor	Retrospective	Average	2000	$0.562	N/A	Transport and travel, salaries and per diems, supervision, training, other campaign-specific costs
		Nationwide SIA in other 16 provinces of Lao PDR					2001	$0.350	N/A	
[36]	Measles vaccine	SIAs conducted in Uganda	Not specified	Not specified	Not specified	Average	2003	$0.531	N/A	Not specified
							2006	$0.686	N/A	
							2009	$0.534	N/A	
[37]	Measles vaccine + VitA	‘Nationwide' SIA -- 50/117 Tanzanian districts	Children, 9–59 mnths	National payer and external donor	Retrospective	Average	2000	$0.312	N/A	Transport and travel, salaries and per diems, supervision and training, other campaign-specific costs (includes social mobilization surveillance)
[38]	VitA + Measles vaccines + Deworming and nutrition screening	Twice-annual Child Health Days in 2/10 Ethiopian regions:	Children, 6–59 mnths	National payer and external donors^(i)^	Retrospective	Average	2006	$0.244	N/A	Training, social mobilization ('sensitization and other promotional meetings'), transport, implementation, evaluation ('review meetings')
		- Amahara								
		- Oromiya					2006	$0.163	N/A	
[39]	Measles vaccine + VitA	Nationwide SIA in Uganda	Children, 6–59 mnths	Not specified	Not specified	Not specified	2001	$0.339	N/A	Not specified
[40]	OPV	National Immunization Day (NID) with costs from 10 provinces in China	Children (Age range not specified)	National payer and external donor^(ii)^	Retrospective	Incremental	1994	$0.657	N/A	Personnel [including per diems for all staff involved in SIA^(iii)^]; publicity and social promotion; training costs, logistical costs (including shipping, freight, fuel, travel cost, vaccine storage, vehicle depreciation, data collection forms)
[41]	Tetanus toxoid vaccine	SIA to provide 3 doses of TT in two phases across 11 'Union Councils' in the district of Loralai, in Baluchistan, Pakistan	Women, 15–45 yrs.	National payer	Not specified	Incremental	2001	$0.491	N/A	Training, social mobilization, per diems, salaries (of health workers, vaccinators, and supervisors), rental of vehicles, fuel, ice for cold chain, monitoring and evaluation, and misc.
[42]	Yellow fever vaccine	SIA in Abidjan and surrounding areas (Anyama, Bingerville and Songon) in Cote d'Ivoire	(I) All unvaccinated children, 6 mnths to 10 yrs.; (II) Persons ≥10 yrs. who are either unvaccinated or whose yellow fever vaccination was >10 yrs. ago^(iv)^	National payer	Retrospective	Average	2001	$0.224	N/A	Transport (inc. fuel and maintenance of rolling stock), administrative/office supplies, social mobilization, human resources, coordination, supervision, cold chain equipment
[43]	Yellow fever vaccine +MenA+C vaccine^(v)^	Reactive immunization campaign in the Matam District, in the Northeast part of Senegal	Children and adults, 1–25 yrs.	National payer	Prospective	Incremental	1997	$0.074	N/A	Staff costs (per diems and travel costs), transport (inc. fuel, maintenance and repairs), cold chain equipment, social/community mobilization, vaccination cards and stationery, coordination and supervision
[44]	MenA+C vaccine^(v)^	Reactive campaign in Réo and Kombissiri districts, Burkina Faso	Children and adults, 2–30 yrs.	National payer, partners, and communities^(vi)^	Prospective	Average	2006	$0.507	N/A	Transportation, personnel, IEC/social mobilization, supervision, surveillance, monitoring & evaluation, training, cold chain equipment and maintenance, waste management, and planning
[45]	MenA+C vaccine^(v)^	Reactive campaign across Guinea in districts with weekly incidence rate (WIR) of menA of >5/100,000	Children and adults, >1 yr.	National payer	Prospective	Incremental	1993	$0.328	N/A	Logistics, wages
[46]	MenA+C vaccine^(v)^	Reactive campaign across Katsina State in Nigeria in districts with WIR of menA of >5/100,001	Children and adults, 6 mnths – 30 yrs.	Not specified	Not specified	Incremental	1996	$0.485	N/A	Transport, personnel, administration
[21]	Cholera vaccine^(vii)^	South Sudanese refugees in Ugandan refugee camps	Children, 1–4 yrs.	National payer	Retrospective	Average	1997	$0.787	N/A	Transport, vaccination cards, storage (free of charge), material for administration, material for data collection, local personnel salaries
[22]	Cholera vaccine^(vii)^	Mass vaccination targeting residents in 13 communes of Hue city, Vietnam	All children and adults, ≥ 2yrs., excluding pregnant women	National payer	Retrospective	Average	1998	$0.142	N/A	Personnel, training, review meeting, and social mobilization (Publicity/information campaign)

^(i)^But excludes UNICEF staff costs, UNICEF and the Federal Ministry of Health’s overhead costs.

^(ii)^Excludes recurrent costs involved in the running of international organizations.

^(iii)^i.e. managers, supervisors, cold-chain technicians, drivers, vaccinators.

^(iv)^Subjects with acute progressive diseases and immune deficiency were excluded.

^(v)^Polysaccharide A + C meningococcal vaccine.

^(vi)^Household costs presented elsewhere.

^(vii)^Oral killed whole-cell/recombinant B-subunit (WC/rBS) cholera vaccine – Not part of existing ADC/E/E programs but included here to provide a more complete picture of literature on SIA operational costs.

Distinction between average and incremental costs is often not made explicit in the literature reviewed. That said, since SIAs tend to be additional to the provision of routine health services, the majority of costs incurred can be considered as incremental rendering these distinctions less relevant. Most of the publications reviewed separated costs into fixed and variable costs, specified the cost components included (albeit using different terminologies and categorizations), and defined the perspective taken [and hence whether economic and/or financial (or accounting) costs, indirect and/or direct costs had been included]. However, studies were less consistent about acknowledging and distinguishing between immunization-specific versus shared costs, and whether/how depreciation for capital equipment was factored into their analyses. In some instances (notably for OPV + measles + VitA SIAs) campaigns were performed in an integrated fashion. In these instances, authors assumed that aside from the costs of vaccines and supplies, other cost components could not be assigned to each antigen/intervention and assumed they would have been the same regardless of whether one intervention or multiple interventions were delivered through the SIA. Variability in these definitions and costing practices makes comparing across the published literature difficult. Furthermore, the information detailed in Table 1 should be read with the above caveats in mind.

### (II)→Analysis of country-specific plans data – See Additional file 2

Given the low number of observations for menA, yellow fever, MR and ‘Other’ SIAs in the dataset, average per person operational costs for these vaccines were not analyzed separately. The observations for these vaccines are included in other aggregate operational cost indicators presented – for the following groups of vaccines:

● Vaccines in GAVI’s portfolio – Based on 51 SIA expenditure observations for measles (n=38), MR (n=3), menA (n=6), yellow fever (n=1), JE (n=2), and typhoid (n=1) vaccines.

● Injectable vaccines – Based on the 51 observations listed above for ‘Vaccines included in the GAVI portfolio’ plus a further 28 observations for tetanus (26), hepatitis B (n=1) and pertussis (n=1) vaccines.

● Orally administered vaccines/interventions – Based on 63 observations for OPV (n=53), vitamin A (n=8) and albendazole/mebendazole (n=2) tablets.

Table 2 summarizes our estimates of (population weighted) average operational costs per person from the cMYP data.

**Table 2:** Weighted Average per person per dose operational costs by vaccine/grouping and size of the country, 2004–2011 – Analysis of cMYP data

**Vaccine/grouping**	**All countries**	**N**	**Small countries (<10 m)**	**N**	**Min ‡**	**Max**^**§**^	**SD**	**Mid-size & large countries (≥10 m)**	**N**	**Min ‡**	**Max**^**§**^	**SD**
OPV	$0.40	53	$0.54	22	$0.14	$1.97	$0.42	$0.39	31	$0.02	$1.77	$0.29
Tetanus vaccines	$0.29	26	$0.80	7	$0.02	$1.83	$0.32	$0.27	19	$0.02	$0.76	$0.13
Measles vaccines	$0.81	38	$1.30	13	$0.27	$3.73	$0.68	$0.79	25	$0.13	$3.55	$0.57
Vaccines in GAVI’s portfolio	$0.98	51	$1.07	17	$0.05	$9.52	$1.44	$0.98	34	$0.03	$3.55	$0.69
Oral vaccines/interventions	$0.39	63	$0.46	27	$0.10	$1.97	$0.42	$0.39	36	$0.004	$1.77	$0.33
Injectable vaccines	$0.77	79	$0.98	24	$0.02	$9.52	$1.45	$0.76	55	$0.02	$3.55	$0.73
All vaccines/interventions		142		51					91			

Additional notes.

N→Number of observations.

SD→Standard Deviation.

‡→Minimum values: In terms of the frequency of occurrence of extremely low values, of the 142 country observations in the dataset, 8% (n=12) of the country observations below $0.10. Seven of these instances related to SIAs for orally administered vaccines/intervention for which we would expect lower costs, and three were for tetanus vaccine SIAs which, on average, have lower operational costs in any case. A sensitivity analysis indicated that removing these observations did not significantly change the findings. While it’s possible that the extremely low values could signify errors in the country plans, these were included in the analyses because they seemed plausible. From an analytic perspective, where low values were observed, we compared these within a countries’ cMYP across diseases, and over time. In the majority of cases where the operational costs were low, the estimates were consistently low suggesting that these were not errors. From a programmatic and budgetary/planning perspective, there are a variety of possible explanations; e.g. reliance on partners or CSOs to conduct (aspects of) the campaign without needing to acknowledge these in the budget; re-programming existing health sector staff to conduct campaign (e.g. making nurses vaccinate children during the SIA) without this shared cost featuring in the immunization budget; reliance on unpaid personnel to conduct campaign (e.g. community health workers to undertake campaign vaccination). It is not uncommon for immunization costing studies to overlook donated costs including that of volunteer time [47].

§→Maximum values: Similarly, while it’s possible that the extremely high values could signify errors in the country plans, these were included in the analyses again because they appeared plausible. 9% of the dataset (n= 13 occurrences) where average operational costs were greater than $1.50 per person. Of these, five were in very small countries (e.g. Cape Verde, Djibouti, Kiribati); two involved vaccines not usually administered through mass vaccination and/or very specific target groups (i.e. typhoid vaccines for food handlers in schools, Hepatitis B targeting special risk groups), and all bar-one were conducted in the African region where per diems often raise the average SIA costs (See below for further details).

### Analysis of cMYP data

Focusing on the results for ‘All countries’ (in Table 2) and outside of OPV/oral interventions and tetanus vaccines, average operational costs per person per dose for this analysis are around US$0.75 to US$1.00. Comparing results in Table 2 versus Table 1 in general, as well as on a country-specific basis (in the penultimate column of Table 1), operational costs from the new analysis of country plans are generally higher than published estimates. Since all cost estimates presented have been adjusted for inflation, the results suggest that costs have increased over time in real terms.

**Figure 2:**
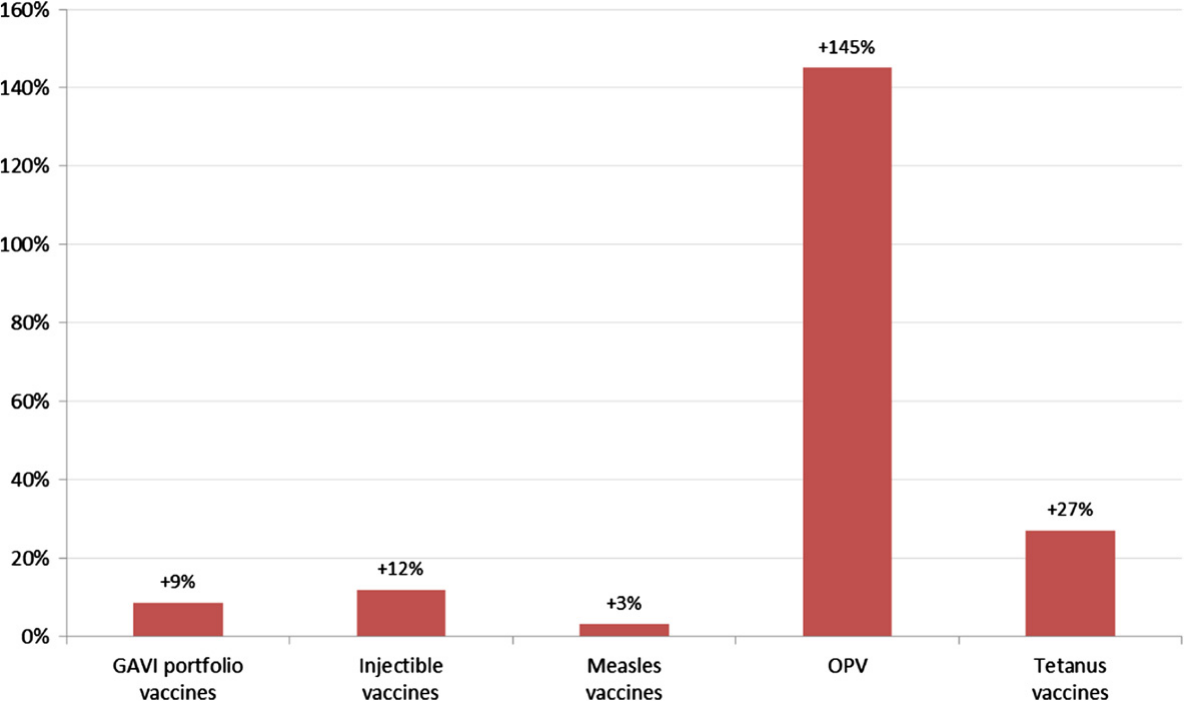
Percentage change in operational costs across the dataset comparing older expenditure estimates (for SIAs conducted between 2004 and 2008) with more recent estimates (for SIAs conducted between 2009 and 2011) – Analysis of cMYP data.

**Figure 3:**
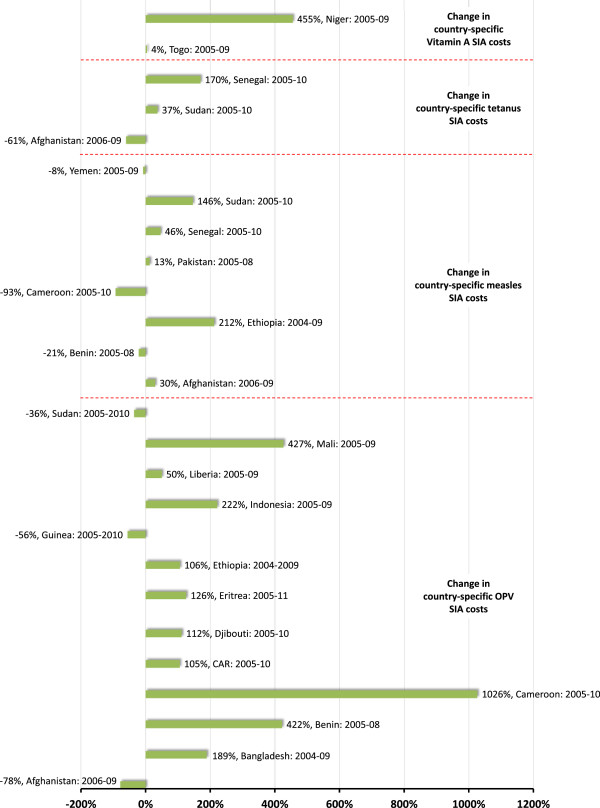
Percentage change in country-specific operational costs (per targeted person per dose) over given time periods – Analysis of cMYP data.

Looking at how operational costs have changed over time *within* the sample of cMYPs themselves, and specifically comparing *in aggregate* more recent operational SIA costs from campaigns conducted over the period 2009–2011 to older estimates from campaigns conducted over the period 2004–2008, we find that across vaccines, more recent campaigns are consistently higher, particularly for OPV SIAs (Figure 2). While pairwise comparisons of operational cost estimates in cMYPs *on a country-by-country basis*, we find that in almost all cases, average per person operational costs have increased markedly, and again particularly for OPV SIAs (Figure 3). One explanation for these increasing trends in costs might be that as the eradication program draws closer to achieving its goal, program officials have increasingly focused resources and efforts on immunizing the hardest (and most costly) to reach and most underserved communities. Of course, since these communities are often at greatest risk of vaccine-preventable disease, these results do not diminish the case for achieving more equitable vaccination coverage.

The analyses also illustrates that average per person per dose operational costs are lower for Midsize/Large Countries than Small Countries, most likely because there are significant economies of scale associated with the conduct of SIAs. Operational costs had a tendency to be lower for OPV and orally administered interventions compared to injectable vaccines, likely explained by the fact that such vaccines/interventions are easier to administer. Personnel delivering these interventions are often ‘lay vaccinators’ (i.e. they may not be skilled health workers qualified to administer injections) and therefore may receive lower salaries, require less training and less costly supervision, all of which can reduce the operational costs.

Operational costs for measles vaccine SIAs are, on average, higher than for OPV and tetanus vaccine SIAs; and since measles vaccine observations dominate the sample used to define operational costs of vaccines in GAVI’s portfolio, these costs are also higher. By contrast, average per person operational costs of tetanus vaccine SIAs appear to be lower than other injectable vaccines. Since cMYP data do not offer breakdowns of cost drivers it is not possible to explain definitively from a programmatic standpoint what might be driving these differences. While further research would be needed to provide a definitive answer, we hypothesize that the lower operational costs associated with tetanus vaccine SIAs may in part relate to the district-specific targeting strategy used in MNT elimination SIAs known as the “high risk approach”– WCBA living in specific high risk districts (HRDs) are targeted with three properly spaced doses of TT [48]. Since these SIAs are almost always conducted sub-nationally in a subset of districts, there is greater ability to work with local antenatal staff and/or local community health workers (CHWs) based in those districts rather than bringing in external vaccinators. Since these local workers generally reside in the community they serve, their proximity to their target population reduces the need for a daily subsistence allowances (“per diems”) over and above basic salaries that would otherwise be factored into operational costs for SIAs conducted nationally. Such localized efforts generally require less extensive supervision, and more modest social mobilization, communication and advocacy (e.g. to publicize efforts on television and radio) efforts.

**Figure 4:**
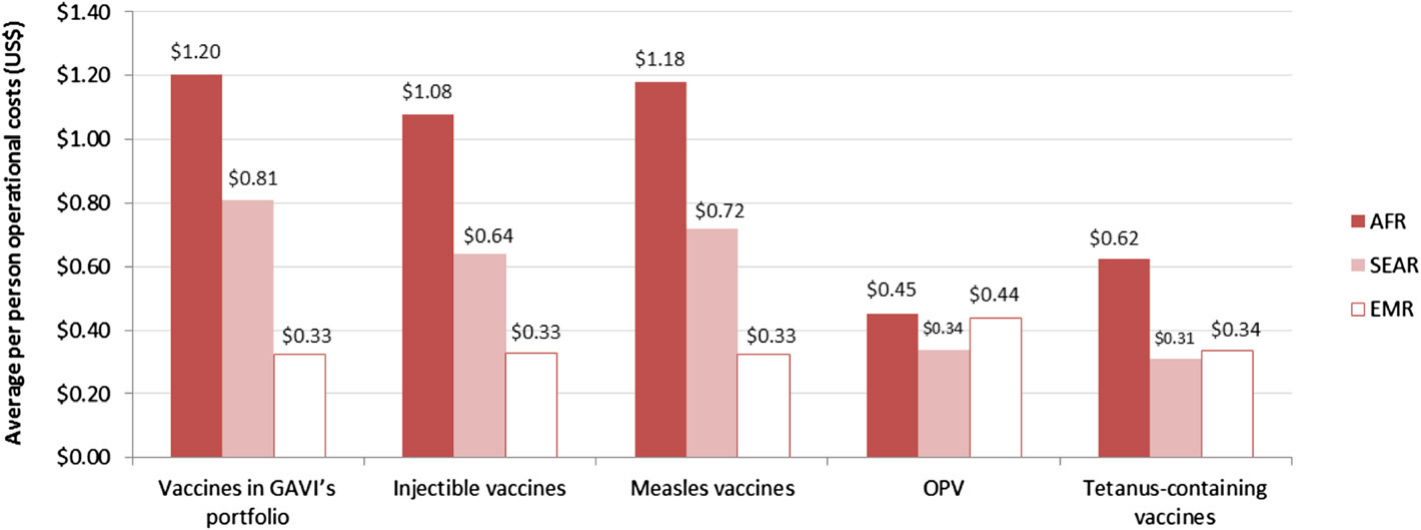
Average operational costs per targeted person per dose (US$) by vaccine grouping and WHO region, 2004–2011 – Analysis of cMYP data.

Average operational costs are consistently higher in the African region (Figure 4) and it is possible that this at least partly relates to a practice most prevalent in AFR, of offering per diems to health workers, particularly for training and outreach activities [49]. However, reaching target populations is generally considered more challenging in AFR as a result of weak infrastructure, and could be a significant factor driving higher operational costs in this region.

**Figure 5:**
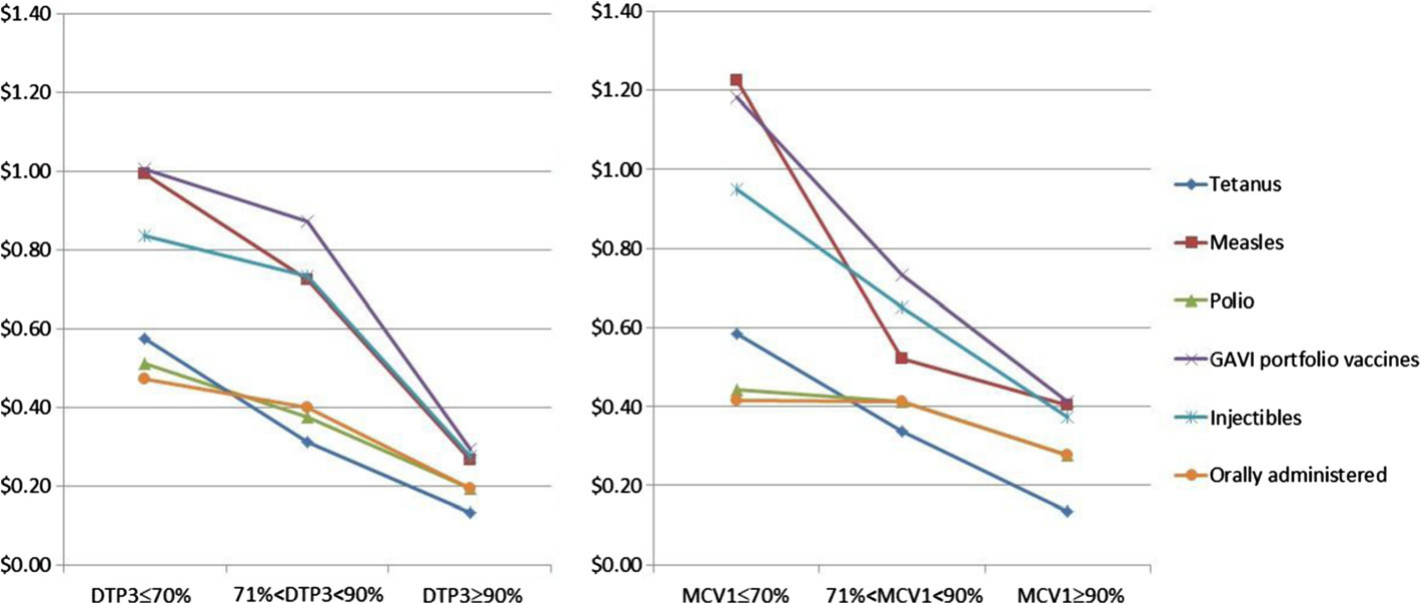
Operational costs per person by vaccine and strength of the routine immunization program (using DTP3 or MCV1 as a proxy for program strength), 2004–2011 – Analysis of cMYP data.

Figure 5 illustrates the average operational costs per person by vaccine grouping and strength of the underlying routine immunization program. Despite the different distributions when using DTP3 or MCV1 coverage as the proxy indicator of system strength (described in the sample characteristics above), both indicators suggest a negative inverse relationship between program strength and operational costs.

### (III)→Analysis of country-specific budgets – See Additional file 2

**Figure 6:**
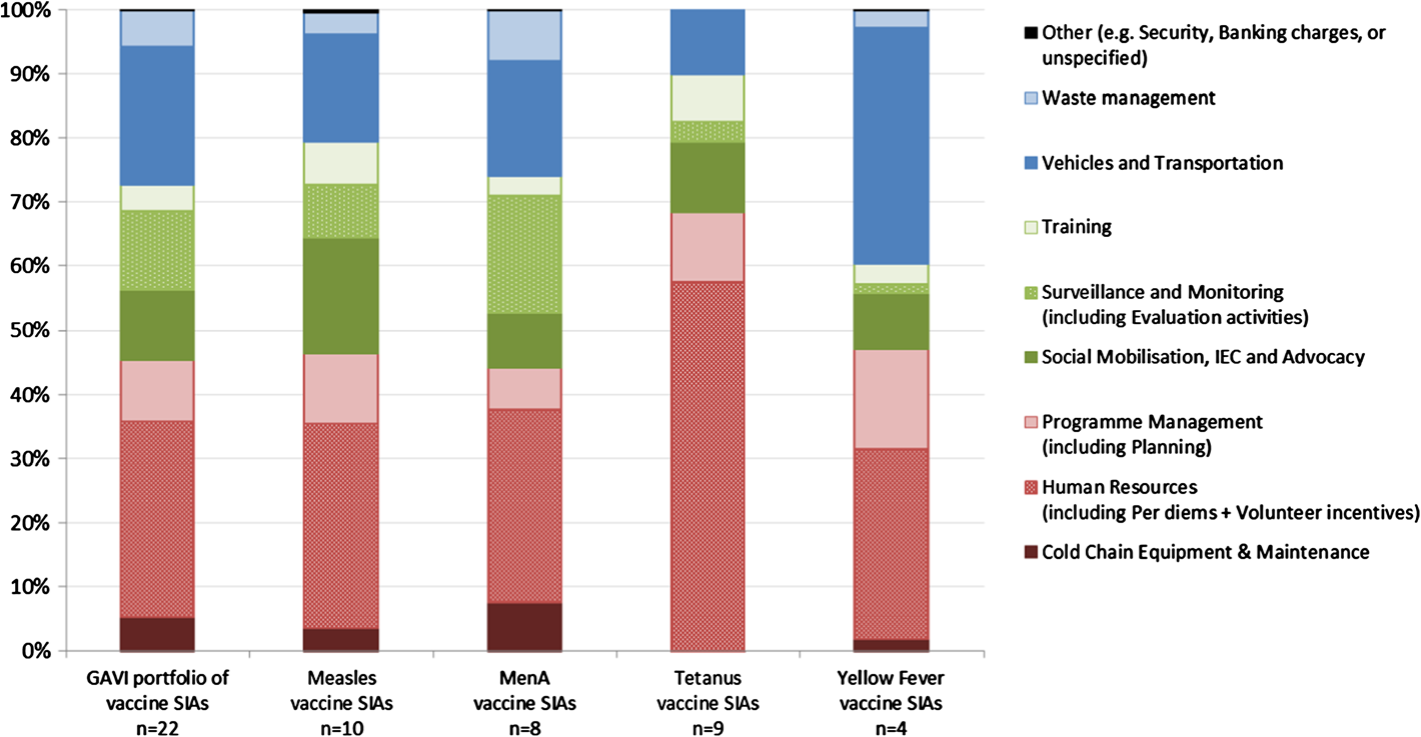
Breakdown of SIA operational costs, 2006–2011 (Relative share in %) – Analysis of SIA budget data.

The breakdown of SIA operational costs by cost component illustrates that four cost drivers make up the largest proportion of costs across all vaccines: human resources (i.e. salaries, per diems), program management, social mobilization (e.g. information, education, communication and advocacy), and vehicles and transportation (e.g. fuel to transport vaccinators). The fact that cold chain equipment makes up such a small proportion of operational costs suggests that SIAs build on existing routine immunization systems and do not require significant additional capital investment (Figure 6).

Compared to other vaccine SIAs, as a proportion of total operational costs Tetanus vaccine SIAs tend to be associated with higher human resources and training costs, and lower vehicles and transportation costs, and virtually no cold chain cost. This finding supports the notion that the HRD approach used in MNT elimination SIAs rely on training local health staff rather than bringing in external vaccinators. While Yellow Fever budget breakdowns also differ from other vaccine SIAs, this variance may be more associated with outliers in the small number of observations in the budget sample.

### (IV)→Limitations

Our analysis of operational costs of SIAs represents a minority of the total SIAs conducted worldwide over the analytical period. For example, our analysis of country-specific plans covered around 11% (41/360) of the measles/rubella-containing vaccine follow-up and catch-up SIAs conducted anywhere in the world between 2004 and 2011 [50]. Since mainly GAVI (eligible and graduating) countries report their immunization expenditures in cMYPs, it is likely that our estimates represent a meaningful summary for this segment of countries, rather than more broadly. Similarly, because cMYPs are not produced by AMR countries, the analysis of country plans is not necessarily reflective of the situation in the Americas. Finally, looking the literature and budget data available, these are heavily skewed towards the AFR region (where the majority of SIAs have historically been conducted) which also limits the applicability of these findings to other regions.

The information documented in both existing literature and possibly the data from cMYPs may not be reflective of the full costs of implementing an SIA. In addition to the operational costs incurred mainly by national payers and reported in the literature and cMYPs, implementation of SIAs that are part of global ADC/E/E efforts often rely on additional expertise from global agents such as WHO, UNICEF, and the United States Center for Disease Control and Prevention (US CDC). These agencies provide highly specialized assistance to governments—undertaking disease surveillance and laboratory testing to monitor epidemiologic dynamics and to help target SIA efforts; developing/implementing social mobilization programs to strengthen community demand for vaccination; and providing other technical support (for example, to bolster program management and training of vaccinators) — ultimately to improve the quality of the campaigns. The costs associated with these additional support activities are often referred to as “*core costs*” in agency budgets but are rarely factored into estimates of operational cost estimates to illustrate the true nature of SIAs^h^.

Finally, the results of the cost analyses presented here are only as strong as the data on which they rest. There are many benefits of cMYPs; e.g., from a global perspective these country plans provide data on immunization program costs (including operational costs of campaigns) across a wide array of countries in a standardized form and using a comparable methodology. However, cMYP data are not systematically validated and may be of variable quality. It is difficult to know what is included in each country’s cMYP estimate of SIA operational costs since breakdowns by cost components are not provided. Thus, despite the availability of guidelines and templates for detailed SIA budgetary planning [51,52], it is possible that variable definitions are used from country to country plan. Looking at the budget data where breakdown are provided, the number of budgets available and analyzed was small. As such, these findings too should be interpreted with care.

## Conclusions

Elimination and eradication of vaccine preventable disease represent the ultimate in addressing health inequities on a local and global scale respectively, while accelerated disease control represents the next best alternative. Achieving ADC/E/E goals is seen as a public health imperative but success since smallpox eradication has been elusive. Robust planning and budgeting, adequate financing, good governance, and effective implementation of SIAs building on strong routine immunization programs are critical to achieving these goals. Our analyses illustrate that existing evidence that often informs budgeting, planning and financing decisions, is out of date, and for many programs, operational costs may be higher than previously thought. Underestimation of costs and funding shortfalls can lead to SIA implementation delays, and can force implementers to target narrower age groups undermining efforts to reach program goals. Therefore, the results of the analysis presented here may provide national and global-level actors with newer SIA operational cost benchmarks to inform planning, budgeting, and financing decisions in absence of better information.

Our analyses also illustrate where program implementers might focus efforts to seek out efficiencies to manage the upward trends in SIA operational costs. For example, with a significant proportion of costs related to human resources, given that there is often criticism of the culture of per diems related to development assistance [53], new assessment of the most cost effective ways to implement SIAs may focus on this and other major cost drivers.

While the information presented here updates the evidence base, there is a need for continued monitoring and additional research into SIA operational costs and expenditures. These efforts might address the deficiencies and discrepancies in monitoring and reporting of operational costs that are highlighted in our review of the literature. Future research might build on the work presented here using more sophisticated multivariate analysis techniques to explore the extent to which each of the factors identified above (e.g. geographic region, population size, breadth of the target population, route of administration, and the strength of the routine immunization program as measured by vaccination coverage) explain and influence operational costs. Future research efforts might also seek to tie operational costs observed to intermediate outcomes such as coverage, or better still final outcomes like confirmed levels of population immunity so as to understand how different levels of investment in SIAs affect outcomes.

There are a number of areas that have yet to be fully explored where future research/monitoring efforts may focus – most notably to tease out the impact on SIA operational costs of routine system strengthening efforts embedded within SIAs. ADC/E/E program strategies are again pointing to the fact that the key to their success lies not just in high-quality and well-funded SIAs, but in effective routine immunization programs built of strong health systems [54]. Creating more robust routine systems requires its own investment increases (P. Lydon, G. Gandhi, J. Vandelaer, J-M Okwo-Bele: *Health systems cost of delivering routine vaccination in low & middle-income countries: What is needed over the next decade?,* Submitted), but where successful, these efforts should provide a broader platform for ADC/E/E programs to reach all communities – ultimately reducing the marginal costs of vaccinating each additional child as illustrated by our findings. By contrast however, there are also calls to exploit the visibility and resources of SIAs in order to strengthen routine immunization (e.g. through training routine health workers, procuring cold chain equipment, and improving injection safety and adverse events management), and there is evidence that such approaches are feasible and can have a positive impact [55-57]. While strengthening routine programs through SIAs could reduce the frequency with which these SIAs are needed, there is anecdotal evidence from measles SIAs (comparing *follow-up* SIAs – where system strengthening efforts are often embedded – to wide age range *catch-up* SIAs where no system strengthening is undertaken) that strengthening routine programs through SIAs will increase the per person costs of those campaigns (Robert Perry: *Personal communication;* 2013).

New research might also focus on the effect of growing security needs on operational costs. Recent attacks on vaccinators, particularly those conducting OPV SIAs in remote areas of the remaining polio endemic countries, are hampering ADC/E/E efforts [58,59]. Additional costs of security for vaccinators in the likes of Northern Nigeria, Pakistan and Afghanistan may drive up SIA costs particularly for SIAs conducted in these areas. Documenting and factoring in these additional costs will be important to ensure SIA budgets are sufficient to overcome these challenges.

Finally, the focus of new studies may need to go beyond existing ADC/E/E programs discussed here. As aforementioned, several underutilized and new vaccines that seem likely to be introduced in the coming years have an SIA delivery component as one-time catch-up strategies, or to periodically maintain herd immunity. Better understanding of the costs of SIAs and how these are likely to change over time will enable more robust assessment of future ADC/E/E policies [60] and help inform the design of new financing strategies to facilitate the large but infrequent investments associated with catch up and follow up SIAs.

## Endnote

^a^Encompassing epidemic prevention.

^b^Operational costs are normally expressed on a “per person” basis. In some instances, these data are expressed on a “per dose” basis. Where there was insufficient information in the paper to express the information on a per person basis, we excluded the paper.

^c^These were the vaccines and programs for which we had sufficient data to draw out vaccine delivery -specific findings; i.e. for menA SIAs targeting the populations below 29 years of age, or yellow fever SIAs targeting those below 60 years of age, there were insufficient observations to assess how operational costs for these vaccines/age groups compared to OPV, measles and TT/Td vaccines.

^d^Since Albendazole/Mebendazole for de-worming and Vitamin A (VitA) are often delivered in campaigns and because many countries have estimated the operational costs of delivering these interventions within their cMYPs, these were included in the per person average costs of orally administered interventions along with OPV.

^e^These population grouping cut-offs were determined based on the countries included in the analysis dataset, balancing a need to ensure sufficient observations in each grouping to explore meaningful differences in operational costs for small versus midsize/large country both by vaccine, route of administration, and overall while simultaneously grouping similar sized countries together (at least where we expect there to be fewest economies of scale—for small countries).

^f^These cut-offs were selected based on published policy/strategy targets associated with the third dose of Diphtheria-Tetanus-Pertussis (DTP3): The GAVI eligibility policy permits countries to apply for most of the routine new/underutilized vaccines so long as they have DTP3 coverage equal to, or greater than 70% [61]. Separately, one of the goals of Global Immunization Vision and Strategy (GIVS) defined in 2005 and reiterated in the DoV GVAP is to reach 90% DTP3 coverage in all national immunization programs [62,63]. However, we also used the same cut-offs when using the first dose of measles-containing vaccine coverage (MCV1) as an alternative proxy for immunization program strength.

^g^As aforementioned, and unpublished analysis of a larger dataset including actual expenditures and projected operational cost data is presented elsewhere (P. Lydon and G. Gandhi: *Introduction of New Vaccines: Analysis of non-vaccine routine and campaign costs for the GAVI Alliance.* Unpublished). The larger dataset covered 257 SIAs and was extracted from 87 unique cMYPs produced by 55 countries – Comparing the results generated from the larger but more heterogeneous dataset of 257 observations with the data/results presented in this paper, the estimates for the former were mostly lower than from the latter.

^h^Although investments to cover core costs are often reported and/or projected by the global coordinators of ADC/E/E programs as part of financial resource requirements (FRRs). [64], (Measles Rubella Initiative: *Measles and Measles-Rubella SIA Forecast 2013–2018.* Unpublished).

## Abbreviations

ADC/E/E: Accelerated disease control, elimination and eradication; SIAs: Supplementary Immunization Activities; MNT: Maternal and neonatal tetanus; menA: *Neisseria meningitis* serogroup A; LICs: Low income countries; MICs: Middle income countries; GPEI: Global Polio Eradication Initiative; MRI: Measles-Rubella Initiative; JE: Japanese Encephalitis; DoV: Decade of Vaccines; GVAP: Global Vaccine Action Plan; US$: United States dollars; cMYPs: comprehensive multi-year plans; OPV: Oral Polio vaccines; TT: Tetanus toxoid; Td: Tetanus-diphtheria; WCBA: Women of child bearing age; VitA: Vitamin A supplement; MR: Measles-Rubella; UNICEF: United Nations Children’s Fund; DTP3: Third dose of Diphtheria-Tetanus-Pertussis vaccines; GIVS: Global Immunization Vision and Strategy; MCV1: First dose of measles-containing vaccines; AFR: African region; EMR: Eastern Mediterranean; SEAR: South East Asia; WPR: Western Pacific; EUR: European; AMR: Americas region; LMICs: Lower Middle Income Countries; CEA: Cost Effectiveness Analysis; WC/rBS: Whole-cell/recombinant B-subunit; HRDs: High risk districts; CHWs: Community health workers; US CDC: United States Center for Disease Control and Prevention.

## Competing interests

The authors declare that they have no competing interests.

## Authors’ contributions

GG participated in the design of the study, carried out the literature review, performed the data analyses, and drafted the manuscript. PL conceived the study, participated in its design, and helped draft the manuscript. Both authors read and approved the final manuscript.

## Pre-publication history

The pre-publication history for this paper can be accessed here:

http://www.biomedcentral.com/1471-2458/14/67/prepub

## Supplementary Material

Additional file 1 **contains three datasets: (1) cMYP data-- the country- reported data extracted from individual cMYPs and adjusted for inflation.** Used for the analysis of operational costs; (2) SIA budget data-- the country-reported data extracted from various sources and adjusted for inflation. Used for the analysis of the cost components that drive SIA costs; (3) Reference-style data—the demographic, immunization system performance, or economic data (e.g. population, vaccination coverage, per capita income, deflators) from third party multilateral sources. Used to categorize country level data and/or create population-weighted average benchmarks. Click here for file

Additional file 2 contains additional summary results and data used in analysis of SIA operation costs and SIA budget breakdowns. Click here for file
